# Hypothalamic Wnt Signalling and its Role in Energy Balance Regulation

**DOI:** 10.1111/jne.12368

**Published:** 2016-03-14

**Authors:** G. Helfer, A. Tups

**Affiliations:** ^1^ Rowett Institute of Nutrition and HealthUniversity of AberdeenBucksburnAberdeenUK; ^2^ Centre for Neuroendocrinology and Brain Health Research CentreDepartment of PhysiologySchool of Medical SciencesUniversity of OtagoDunedinNew Zealand

**Keywords:** Wnt signalling, hypothalamus, energy homeostasis, glucose homeostasis, photoperiod

## Abstract

Wnt signalling and its downstream effectors are well known for their roles in embryogenesis and tumourigenesis, including the regulation of cell proliferation, survival and differentiation. In the nervous system, Wnt signalling has been described mainly during embryonic development, although accumulating evidence suggests that it also plays a major role in adult brain morphogenesis and function. Studies have predominantly concentrated on memory formation in the hippocampus, although recent data indicate that Wnt signalling is also critical for neuroendocrine control of the developed hypothalamus, a brain centre that is key in energy balance regulation and whose dysfunction is implicated in metabolic disorders such as type 2 diabetes and obesity. Based on scattered findings that report the presence of Wnt molecules in the tanycytes and ependymal cells lining the third ventricle and arcuate nucleus neurones of the hypothalamus, their potential importance in key regions of food intake and body weight regulation has been investigated in recent studies. The present review brings together current knowledge on Wnt signalling in the hypothalamus of adult animals and discusses the evidence suggesting a key role for members of the Wnt signalling family in glucose and energy balance regulation in the hypothalamus in diet‐induced and genetically obese (leptin deficient) mice. Aspects of Wnt signalling in seasonal (photoperiod sensitive) rodents are also highlighted, given the recent evidence indicating that the Wnt pathway in the hypothalamus is not only regulated by diet and leptin, but also by photoperiod in seasonal animals, which is connected to natural adaptive changes in food intake and body weight. Thus, Wnt signalling appears to be critical as a modulator for normal functioning of the physiological state in the healthy adult brain, and is also crucial for normal glucose and energy homeostasis where its dysregulation can lead to a range of metabolic disorders.

## Introduction

In this review, we discuss the importance of Wnt signalling, which has recently emerged as a key player in glucose and energy homeostasis in the hypothalamus of mammals, and we focus on various aspects of hypothalamic Wnt signalling in obese and seasonal models.

First discovered in *Drosophila*, the Wnt signalling pathway was called ‘wingless’ (Wg) because its mutation led to a loss of wing tissue 1. In mice, it was reported to promote tumour formation and was termed ‘integration‐1’ (int1) 2. Wingless and int1 were later combined to ‘Wnt1’ because they are evolutionary highly conserved and the large family of related genes is now commonly known as Wnt (short for wingless‐related integration site) genes, part of the Wnt signalling pathway 3.

**Figure 1:**
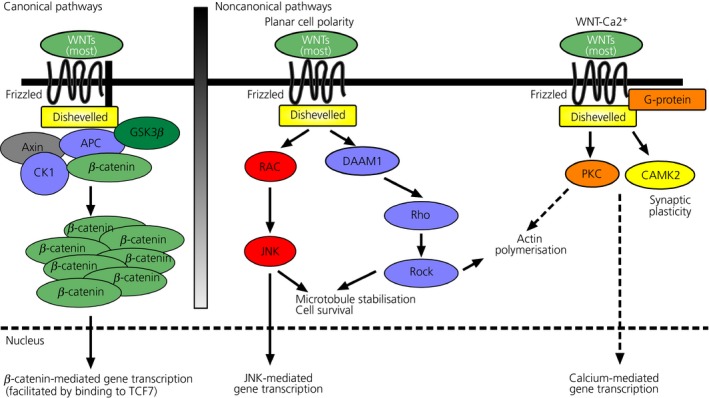
Overview of the Wnt signalling pathways. Wnt signalling occurs as (a) the canonical, (b) planar cell polarity (PCP) and (c) Wnt/Ca^2+^ pathways. APC, adenomatous polyposis coli; Axin, Axis‐inhibition protein; CAMK2, calcium/calmodulin‐dependent protein kinase II; CK1, casein kinase 1; DAAM, dishevelled‐associated activator of morphogenesis; GSK3, glycogen synthase kinase 3; JNK, c‐Jun N‐terminal kinase; NFAT, nuclear factor of activated T cells; PKC, protein kinase C; ROCK, Rho‐associated protein kinase; TCF7, Transcription factor 7.

To date, 19 Wnt genes have been described that encode for peptides and proteins acting in an autocrine/paracrine manner and these often have overlapping or redundant functions 4. These Wnts are involved in three different pathways, known as the canonical β‐catenin dependent signalling pathway and two noncanonical β‐catenin independent pathways leading to signal transduction via c‐jun N‐terminal kinase (JNK) or Ca^2+^. Many Wnt proteins, such as Wnt1, Wnt3a and Wnt10b, are involved in the canonical β‐catenin dependent pathway, resulting in β‐catenin activation, whereas only a few Wnt proteins (e.g. Wnt5a, Wnt11) are involved in the two β‐catenin independent pathways (Fig. 1). The complex Wnt pathways have been reviewed extensively recently 4, 5, 6, 7 and we only provide a short summary here before discussing aspects of Wnt signalling in the adult hypothalamus and energy balance regulation.

## A brief overview of the Wnt signalling pathways

The canonical β‐catenin dependent pathway is well characterised and is known for its involvement in the regulation of cell differentiation and cell proliferation 4, 5. The main biological effect is to regulate β‐catenin stabilisation by glycogen synthase kinase 3β (GSK3β). Canonical Wnt/β‐catenin signalling is activated when a canonical Wnt ligand binds to a frizzled (Fzd) receptor, which then forms a complex with the co‐receptor lipoprotein receptor‐related protein (LRP) 5/6. This leads dishevelled (Dvl) to phosphorylate LRP, thereby inactivating GSK3β. GSK3β inactivation in turn decreases phosphorylation of the transcriptional co‐activator β‐catenin. Stabilised cytoplasmatic β‐catenin can then enter the nucleus where it associates with transcription factors of the lymphoid enhancer factor (Lef)/T cell factor (TCF) family to regulate the transcription of downstream target genes such as cyclin D1 and axin 2 4, 5, 6. Additionally, several regulators of the Wnt pathway have been identified, some act by inhibiting the interaction of Wnt ligands with the Fzd receptors, such as members of the secreted frizzle‐related protein family (sFrp), and others interfere with the formation of an active Fzd receptor‐LRP complex, such as Dickkopf (Dkk) 7.

In the noncanonical pathways, Wnts function independently of β‐catenin. Similar to the β‐catenin dependent pathway, Wnt ligands bind to Fzd receptors forming a complex with a co‐receptor. This enables the planar cell polarity (PCP) pathway to activate JNK. The downstream output of the PCP/JNK pathway regulates cytoskeletal reorganisation and can lead to changes in cell polarity by directing Dvl signals to the monometric GTPases Rho and Rac, which stimulate Rho‐associated protein kinase and JNK, respectively 4. The second β‐catenin independent pathway, which was first described in zebrafish (*Danio rerio*) and *Xenopus laevis* embryos, is the Wnt/Ca^2+^ pathway 8. Here, Wnts trigger Fzd‐mediated activation of G‐protein, which in turn up‐regulates phospholipase C to increase intracellular Ca^2+^ and diacylglycerol. This stimulates protein kinase C and the rise in intracellular Ca^2+^ that enables CamKII and calcineurin to modulate transcription through the regulation of cAMP response element‐binding protein and nuclear factor of activated T cells. The Wnt/Ca^2+^ pathway is prominently involved in cancer, inflammation and neurodegeneration 4.

The three Wnt pathways do not function autonomously; rather, they can act in concert with many Wnt ligands and their downstream targets are often involved in more than one pathway, which often regulate each another 6 (Fig. 1).

## Evidence for Wnt signalling in the developed hypothalamus

Wnt proteins have been considered as important mediators of cell–cell communication and play a prominent role in diverse cellular processes ranging from cardiovascular physiology to cancer metabolism 3, 9. In the nervous system, the Wnt pathway has been predominantly characterised in embryonic development. Within the past decade, it became clear that it also plays a major role in the adult brain morphogenesis and function. To date, Wnt signalling has only been described in a limited number of brain regions and brain functions, with studies mainly focussing on the hippocampus. The hippocampus is important for learning and memory, and a rich literature now links the family of Wnt genes with hippocampal plasticity and memory formation 5. Recent data indicate that Wnt signalling also requires investigation in the hypothalamus, the brain centre that is key in energy balance regulation 10. Here, Wnt signalling was initially also studied in the context of early neural development where its importance was shown *in vitro* in hypothalamic neuropeptide Y (NPY) cell lines and *in vivo* in mouse and zebrafish hypothalamic development 11, 12, 13, 14, 15. Using Lef1 loss‐of‐function zebrafish embryos, Lee *et al*. 11 were able to demonstrate that the transcriptional activator Lef1 is essential during the development of the posterior hypothalamus in zebrafish, and also that it signals through the canonical Wnt pathway in this brain region. Subsequently, it was demonstrated that Wnt signalling in the hypothalamus is required for the differentiation of neural progenitors during the entire lifespan in zebrafish 16, 17 and this is evolutionary conserved throughout the vertebrate subphylum because Wnt signalling is also essential in the hypothalamus of mice, where it acts as an inhibitor of radial glia differentiation 17. These data suggested that Wnt signalling required a more careful consideration in the adult hypothalamus.

**Figure 2:**
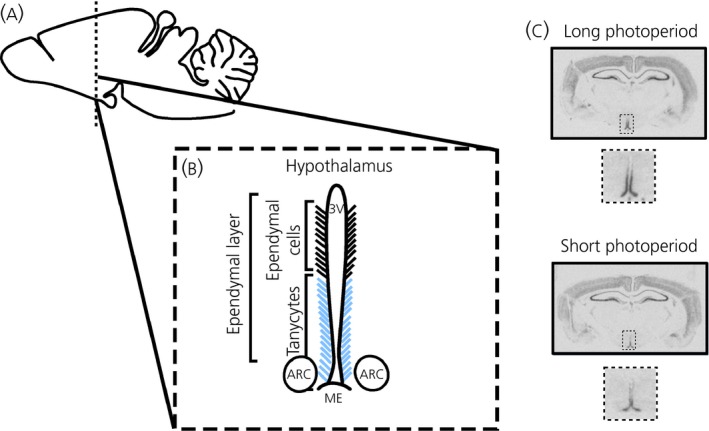
The neuroanatomical location of Wnt signalling genes in the hypothalamus. (a) Overview of a rat brain. The dotted lines indicate the position of the coronal section in (b). (b) Diagram of a rat brain, detailing the hypothalamic region that expresses Wnt signalling genes. All of the Wnt genes that have been investigated so far are expressed in the ependymal layer lining the third ventricle and the arcuate nuclei. (c) Wnt signalling genes are expressed in the brain of F344 rats, including the cortex, hippocampus and hypothalamus. The coronal sections show the expression of the antagonist Dickkopf 3 (Dkk3) in the ependymal layer as a representative example for many Wnt molecules. *Dkk3* mRNA was detected by *in situ* hybridisation with antisense ^35^S‐labelled riboprobes in F344 rats held under long photoperiod or a short photoperiod for 4 weeks. Magnifications show a detailed view of the hypothalamic regions indicated in (b). ARC, arcuate nucleus; ME, median eminence, 3V, third ventricle.

One of the first indications that Wnt signalling is important in the developed mammalian hypothalamus emerged from microarray studies conducted in seasonal mammals, which identified differential gene regulation of components of the Wnt pathway in response to photoperiod (day length) in key regions of energy balance regulation 18. At the same time, the mediobasal hypothalamus of adult mice was found to contain a Wnt‐responsive cell population 17 and reports in diet‐induced and genetically obese (leptin deficient) mice indicated a critical role for hypothalamic Wnt signalling in the energy and glucose metabolism of mammals 19, 20. Key loci for Wnt signalling within the hypothalamus are the tanycytes and ependymal cells lining the third ventricle and the arcuate nucleus neurones, which comprise an important centre of body weight regulation because these cells express all of the Wnt signalling genes (i.e. from ligands to target genes) 17, 20, 21 (Fig. 2). Recent work from divergent fields has emphasised the importance of tanycytes and ependymal cells in neuroendocrine function. From an appetite and energy balance perspective, it has become evident that they represent a stem cell population that gives rise to the appetite regulatory neurones essential for metabolic regulation, whereas research in seasonal biology has shown that these cells are crucial for long‐term changes in metabolic physiology 22.

## The neuroendocrine role of Wnt signalling in glucose and energy homeostasis

Recent work has highlighted a critical role for Wnt signalling in the neuroendocrine control of glucose and energy metabolism of mammals, where its dysregulation has been profoundly implicated in metabolic disorders such as type 2 diabetes and obesity. An initial, striking link between Wnt signalling and metabolic impairments was established by genome‐wide association studies in humans that linked the transcriptional co‐activator of β‐catenin, TCF7/L2, with a higher risk of developing type 2 diabetes 23, 24, 25. Further indications linking the Wnt signalling system with glucose homeostasis were provided by *in vitro* studies showing that the Wnt/β‐catenin pathway is responsive to glucose 26. In addition, GSK3β, an endogenous inhibitor of the canonical Wnt signalling pathway, potently affects energy and glucose homeostasis in a diet‐sensitive manner 19.

**Figure 3:**
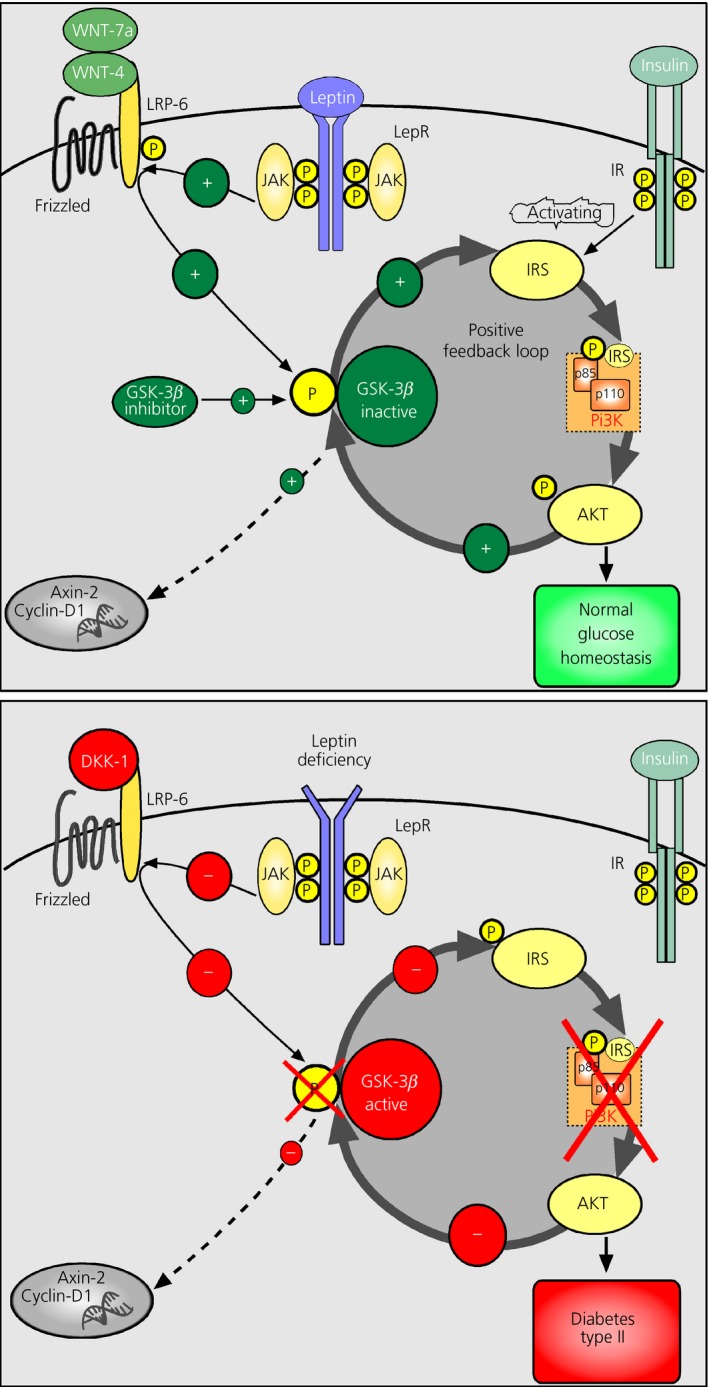
Model of Wnt signalling in energy balance regulation. (a) Leptin activates lipoprotein receptor‐related protein (LRP)‐6, which results in inactivation of glycogen synthase kinase 3β (GSK3β). This might trigger a positive‐feedback loop in which the phosphorylation by GSK3β on inhibitory phosphorylation sites of insulin receptor substrate 1 (IRS‐1) is reduced. The modification of IRS‐1 might result in an activation of the IRS‐phosphoinositide 3‐kinase (PI3K) pathway by insulin increasing phospho‐AKT. Phospho‐AKT then might enhance this mechanism through inactivation of GSK3β. (b) By contrast, in genetically obese leptin‐deficient mice, there is no inactivation of LRP‐6 by leptin. Therefore, increased GSK‐3β activity inhibits IRS‐1 through phosphorylation of inhibitory sites. This inhibition can potentially lead to hypothalamic insulin resistance and subsequently to the development of type‐2 diabetes. Axin‐2, Axin inhibition protein 2; DKK1, Dickkopf 1; LepR, leptin receptor; IR, insulin receptor; JAK, Janus kinase. Reprinted with permission 20.

Because, within the brain, it is the hypothalamus that is critical in sensing and integrating peripheral signals 10, Wnt signalling molecules were investigated in hypothalamic regions that play a role in metabolic homeostasis 19, 20. Although the expression of Wnt signalling genes was found throughout the brain, not surprisingly including the hippocampus 5, most genes were also strongly expressed in the mediobasal hypothalamus containing the arcuate nucleus, the appetite‐regulating centre 20 (Fig. 2 and Table 1). It is well established that the arcuate nucleus contains cell populations that are leptin‐responsive, with leptin inhibiting orexigenic Agouti‐related peptide/NPY neurones and activating anorexigenic pro‐opiomelanocortin (POMC) neurones 10. In leptin‐deficient mice, Wnt signalling was impaired in the mediobasal hypothalamus. The expression of Wnt ligands, Wnt7a and Wnt4, as well as the Wnt target genes, axin‐2 and cyclin D1, was lower in Lep^ob/ob^ mice compared to Lep^+/+^ mice (Table 1). This effect was dependent on the lack of leptin because leptin replacement restored gene expression. Furthermore, pharmacological blockade of Wnt/β‐catenin signalling with the endogenous Wnt inhibitor Dkk1 prevented the ability of leptin to improve glucose homeostasis 19, 20. Leptin activates Wnt signalling via GSK3β inhibition in leptin responsive NPY neurones, suggesting that the catabolic action of the hormone is mediated via hypothalamic WNT signalling 19, 20. The idea that the Wnt pathway represents a novel integration site for the leptin signal is further confirmed by recent finding that leptin administration acutely leads to the activation of LRP‐6 in hamsters and mice 20, 27. Additional studies are required to clarify whether leptin directly activates the Wnt co‐receptor (e.g. through interaction with leptin receptor associated Janus kinase) or whether the activation is indirect and distal to the leptin receptor. The very rapid activation of LRP‐6 (within 15 min after i.p. leptin administration) points toward a direct action of leptin on the Wnt pathway (Fig. 3).

**Table 1:** Summary of Hypothalamic Wnt Signalling Genes in Different Model Organisms and the Known Stimuli That Cause Gene Expression Changes

Functional gene grouping	Gene name	Species	Stimulus for gene expression change if known	Reference
Wnt/β‐catenin pathway				
Axin‐1	Axin‐1	Mouse		20
TCF‐7	T‐cell specific transcription factor 7	Mouse		20
β‐Cat	β‐catenin	Mouse		20
DSL	Dishevelled	Mouse		20
		Rat	LP↑	44
Fzd5	Frizzled homologue 5	Mouse		20
GSK‐3β	Glycogen synthase	Mouse	Leptin↑ HFD↑	19, 20
	kinase 3β	Hamster		27
Lef 1	Lymphoid enhancer binding factor 1	Rat	LP↑	44
Fzd9	Frizzled homologue 9	Rat	LP↑	44
Tcf‐3	Transcription factor 3	Rat	LP↑	44
Wnt/Ca^2+^ pathway/ligands				
Wnt4	Wingless‐type 4	Mouse	Leptin↑	20
		Hamster	LP↑	27
Wnt7a	Wingless‐type 7a	Mouse	Leptin↑	20
Wnt9a	Wingless‐type 9a	Rat	LP↑	44
Wnt9b	Wingless‐type 9b	Rat	LP↑	44
Wnt signalling regulation				
Dkk1	Dickkopf 1	Mouse	Leptin↑	19
Dkk3	Dickkopf 3	Mouse		20
		Rat	LP↑ NMU↑	21, 44
		Hamster		27
sFrp2	Secreted frizzle related protein 2	Rat	LP↑ NMU↑	21, 44, 45
		Hamster	LP↑	27
Mfrp	Membrane frizzle related protein	Rat	LP↑	45
Wnt‐signalling target genes				
Axin‐2	Axis inhibition protein 2	Mouse	Leptin↑	20
		Hamster		27
CCND1	Cyclin D1	Mouse	Leptin↑	20
		Hamster		27

HFD, high‐fat diet; LP, long photoperiod; NMU, neuromedin U.

In line with this evidence, targeted deletion of β‐catenin in the hypothalamus of mice also had direct effects on NPY and POMC neurones, leading to a reduction in postnatal body weight compared to control mice 28, which suggests that the Wnt pathway regulates obesity by controlling food intake behaviour. In this context, it is also interesting to note that challenges to metabolic homeostasis via energy restriction alter the expression of genes associated with Wnt signalling in the mouse hypothalamus in an age‐dependant manner 29.

## Wnt pathway in whole body metabolism

In addition to the neuroendocrine role of Wnt signalling in centrally controlled energy balance regulation, recent evidence suggests that Wnt/β‐catenin signalling also regulates whole‐body metabolism, acting on peripheral organs and tissues involved in systemic energy homeostasis. For example, inactivation of β‐catenin in the murine liver has been implicated in the development of diet‐induced fatty liver disease, obesity and systemic insulin resistance 30. In the pancreas of adult mice, targeted deletion of β‐catenin leads to glucose intolerance and protection from diet‐induced obesity and insulin resistance 31.

It is now well recognised that adipose tissue is crucial for the regulation of energy homeostasis 32. A number of studies have suggested that components of the Wnt/β‐catenin pathway play an important role in adipocyte function and adipogenesis by inhibiting the differentiation of adipose tissue progenitor cells 33, 34, 35, 36 and this has been implicated in the control of adipose tissue with respect to the regulation of glucose homeostasis and the development of obesity 37, 38, 39, 40. The importance of Wnt activation in adipogenesis has mainly been supported by data obtained *in vitro*, although it has also been confirmed by an *in vivo* study showing that adipocyte‐specific over‐expression of Wnt10b in Lep^ob/ob^ mice stabilises β‐catenin, thereby reducing adiposity and improving insulin sensitivity 35, 36.

All of these studies have focused on the β‐catenin dependent Wnt pathway in adipose tissue biology and energy homeostasis. A very recent study has shown that the noncanonical Wnt pathways also play an essential role in obesity and metabolic dysfunction by increasing adipose tissue inflammation. Fuster *et al*. 41 showed that over‐expression of Wnt5a, a Wnt protein associated with the noncanonical pathway 42, increases adipose tissue inflammation, leading to a greater impairment of glucose homeostasis. Conversely, Wnt5a ablation in diet‐induced obese mice reduces adipose tissue inflammation and thereby insulin resistance. The effects of Wnt5a are independent of adipogenesis or adipose tissue expansion 41.

Interestingly, changes in adipose Wnt signalling can precede adiposity, as demonstrated in an elegant study investigating the epigenetic mechanisms involved in obesity 43. Wnt signalling genes regulate adipose tissue expansion in mice with low and high weight gain and this can even occur before mice are fed a high‐fat diet. In addition to the effect of Wnt signalling genes on adipose tissue, gene expression was also compared in the brain of low and high weight gaining mice, although differences in Wnt molecules in the hypothalamus were not detected 43.

## Wnt signalling in seasonal animals

Together, these data provide persuasive evidence for a significant role of Wnt signalling in glucose homeostasis and energy balance regulation in abnormal physiological states. It is clear that aberrant Wnt signalling can lead to a range of metabolic disorders. Much less common are reports of the Wnt signalling pathway as a modulator of the physiological state in the healthy adult brain. Yet, recent evidence has shown that the Wnt signalling pathway in the hypothalamus is regulated not only by diet and leptin, but also by photoperiod (day length) in seasonal mammals 18, 21, 44, 45, which is connected to natural adaptive changes in body weight and food intake 46. Mammalian species sensitive to photoperiod changes, such as the Djungarian (Siberian) hamster (*Phodopus sungorus*), the common vole (*Microtus arvalis*), the F344 rat strain and the CBA/N mouse strain, are important animal models for studying long‐term physiological changes in energy balance, in which a simple change in the environmental photoperiod cue induces dramatic and robust alterations in food intake, body weight and body composition. In the examples noted above, short (winter‐like) photoperiods lead to a decrease in food intake and body weight, whereas, in long (summer‐like) photoperiods, food intake and body weight are increased.

Although laboratory rats are generally not responsive to photoperiod, the juvenile F344/NHsd rat is one of the few strains of rats that has retained its photoperiodic sensitivity in terms of reproductive function, growth and metabolism 45, 47, therefore providing a useful system for dissecting pathways that regulate photoperiodic gene expression in the hypothalamus. Genome‐wide expression analyses comparing these two natural states in the photoperiod‐sensitive F344 rat indicate that, in addition to the well‐characterised photoperiod induced changes in thyroid hormone signalling gene expression 48, the Wnt/β‐catenin signalling pathway is another major pathway altered in response to photoperiod 18. Subsequently, a whole range of Wnt related genes are described as being up‐regulated by a long photoperiod and these are predominantly members of the canonical Wnt/β‐catenin pathway 21, 44, 45 (Table 1). These changes, however, are independent of changes in local hypothalamic thyroid hormone signalling, a potent driver of seasonal body weight regulation, because central administration of thyroid‐stimulating hormone does not alter the expression of Wnt‐signalling genes in the ependymal layer of F344 rats 44. This is interesting given that most seasonal research focuses on the importance of thyroid hormone driving seasonal energy balance regulation, emphasising that other pathways might play an equally important role in this process 48.

In the F344 rat, the Wnt signalling genes are predominantly expressed in the ependymal cells and tanycytes lining the third ventricle of the hypothalamus 21 (Fig. 2). A similar photoperiodic response of Wnt pathway molecules has been confirmed in the hypothalamus of Djungarian (Siberian) hamster, although here abundant expression occurs for most genes in the arcuate nucleus 27. In addition to a species‐specific expression pattern, the anatomical location for individual Wnt genes also appears to be heterogeneous. For example, in the hamster, Wnt7a is expressed in the lateral part of the arcuate nucleus towards the border of the lateral hypothalamus, whereas Dkk3 is mainly expressed medially in the tanycytes 27. The observation that the Wnt components are not expressed uniformly in hypothalamic nuclei suggests that individual components of the system might play different roles in different locations.

In conjunction with the findings described in obese models, it is reasonable to speculate that the photoperiod‐induced changes in the Wnt pathway are also directly related to changes in glucose and energy homeostasis. Some support for this hypothesis may be evident from the finding that neuromedin U (NMU), a neuropeptide that has been linked to acute energy balance regulation 49, increases mRNA expression of two regulators of the canonical Wnt pathway, sFrp2 and Dkk3, in the ependymal layer of F344 rats 44. However, intracerebral injections of NMU rapidly decreased food intake and body weight and increased energy expenditure and thermogenesis in seasonal and obese model systems 44, 49, 50, 51, 52. These rapid changes associated with acute energy balance regulation do not comply with the long‐term changes in seasonal animals, where long photoperiod, which is associated with an increase in food intake and body weight, increases Wnt signalling pathway genes (Table 1).

A hypothesis more consistent with the longer‐term physiological changes characteristic of seasonal animals would be that seasonal changes in Wnt signalling gene expression might contribute to the cellular and structural remodelling of the hypothalamic networks involved in the control of seasonal energy balance. Studies conducted in the hamster and sheep suggest that morphological changes in the mediobasal hypothalamus are associated with changes in photoperiod 53, 54, 55, 56. In the F344 rat, cell proliferation in the hypothalamus is also under seasonal control 57, suggesting that some form of structural and cellular re‐modelling may be part of the neuroendocrine mechanism involved in the seasonal regulation of energy balance.

As described earlier, the Wnt pathway has been strongly implicated in synaptic maintenance and function, as well as adult neurogenesis. Importantly, hypothalamic neurogenesis appears to be significant in the regulation of multiple neuroendocrine pathways, such as in the control of feeding behaviour and body weight regulation 58, 59, 60, 61. Consistent with this idea, Wnt signalling regulates hypothalamic progenitor differentiation in the zebrafish 17. Tanycytes are essential for neuroendocrine function in the hypothalamus, and these radial glial cells also serve as a neural progenitor population 22. It is primarily the tanycytes that express the Wnt signalling genes in the F344 rat in a photoperiodic manner. Interestingly, the tanycytes also express retinoic acid signalling components 21, 57, 62 and, in the hippocampus of the adult mouse where adult neurogenesis has been confirmed, members of the Wnt gene family are downstream components of retinoic acid‐regulated neurogenesis 63. Furthermore, the G protein coupled receptor 50 (GPR50), a melatonin‐related receptor, appears to promote neuronal differentiation of embryonic neuronal progenitor cells through canonical Wnt signalling 64. This is significant because GPR50 is expressed in tanycytes 65 adjacent to or overlapping with Wnt signalling genes 20, 21 and knockout of GPR50 in mice is functionally linked to energy homeostasis leading to an aberrant leptin response 66, 67.

However, in contrast to this hypothesis, Wang *et al*. 17 found that Wnt pathway activation leads to a decrease of tanycyte numbers in the mouse hypothalamus, suggesting that tanycyte formation and maintenance does not necessarily require Wnt signalling in mammals, thus adding yet another level of complexity. Therefore, the biological role of Wnt signalling and its potential role in hypothalamic neurogenesis in seasonal mammals remains a fascinating avenue for further research.

## Concluding remarks

Collectively, several studies conducted in seasonal and obese models have revealed a specific role for members of the Wnt signalling family in energy balance regulation. Based on scattered findings that report the presence of Wnt molecules in the hypothalamus and indicate their potential importance in key regions of food intake and body weight regulation 17, 18, work undertaken in our laboratories has begun to follow up the individual Wnt genes involved in this system and decipher their relevance. It is clear that hypothalamic Wnt signalling is a critical signalling pathway utilised by leptin, and appears to be a key mechanism providing convergence in the actions of leptin and insulin 19, 20. However, research on this topic is still in its infancy, and considerably more work is required to clarify the molecular mechanisms involved and its precise biological significance.

Nevertheless, these findings have now opened the field to future studies investigating the central role of this process in the neuronal control of metabolism. The hypothalamus of seasonal mammals comprises a strong model for studying Wnt function in the healthy brain, whereas targeting Wnt signalling pathways in diet‐induced and genetically obese models can potentially lead to therapeutic benefits for metabolic disorders such as diabetes and obesity in humans.
